# Prevalence and factors associated with body image dissatisfaction among school student: National School Health Survey, 2019

**DOI:** 10.1590/S2237-96222024v33e20231441.en

**Published:** 2024-12-06

**Authors:** Ingride Sousa Linhares, Osmar de Oliveira Cardoso, Jesusmar Ximenes Andrade, Fernando Ferraz do Nascimento, Malvina Thais Pacheco Rodrigues, Márcio Dênis Medeiros Mascarenhas

**Affiliations:** 1 Universidade Federal do Piauí, Programa de Pós-Graduação em Saúde e Comunidade, Teresina, PI, Brazil

**Keywords:** Adolescente, Imagen Corporal, Salud, Instituciones Académicas, Estudios Transversales, Adolescent, Body Image, Health, Schools, Cross-Sectional Studies

## Abstract

**Objective:**

To estimate prevalence and analyze factors associated with body image dissatisfaction among Brazilian adolescent school students.

**Methods:**

Cross-sectional study with data from the 2019 National School Health Survey (PeNSE). Prevalence of self-reported body image dissatisfaction and respective 95% confidence intervals (95%CI) and its association with individual characteristics were estimated via odds ratios (OR) and 95% CI using logistic regression.

**Results:**

Of the 159,245 students, 30.2% (95%CI 29.2;31.1) reported body image dissatisfaction, which was associated with all factors analyzed. Likelihood of dissatisfaction was greater among females (OR = 3.86; 95%CI 3.45;4.32), having internet at home (OR = 8.68; 95%CI 6.83;11.03), thinking that no one cares about them (OR = 3.02; 95%CI 2.60;3.50), that life is not worth it (OR = 3.27; 95%CI 2.88;3.72) and feeling irritated (OR = 2.87; 95%CI 2.53;3.26).

**Conclusion:**

Body image dissatisfaction is associated with various factors and requires an intersectoral approach.

## Study contributions

**Main results:**

Prevalence of body image dissatisfaction was 30.2%. The greatest likelihood of dissatisfaction was more related to being female, having internet at home, frequently feeling that no one cares about them, that life is not worth it, and feeling irritated.

**Implications for services:**

We suggest that projects be carried out in schools encouraging physical activity, less screen time and good eating habits, in addition to providing psychological support to students, in order to guide care for their mental health.

**Perspectives:**

Projects are needed to encourage and instruct good physical and psychological health practices at school, involving the entire school community, training of education professionals and encouragement of adolescent mental health self-care.

## RESUMEN

**Objetivo:**

Evaluar la prevalencia y analizar factores asociados a la insatisfacción con la imagen corporal en adolescentes brasileños.

**Métodos:**

Estudio transversal con datos de la Encuesta Nacional de Salud Escolar (PeNSE) de 2019. La prevalencia de insatisfacción autorelatada con la imagen corporal y sus respectivos intervalos de confianza del 95% (IC95%) y su asociación con las características individuales se estimaron mediante el odds ratio (OR) y el IC95% mediante regresión logística.

**Resultados:**

De los 159.245 estudiantes, el 30,2% (IC_95_% 29,2;31,1) refirió insatisfacción con la imagen corporal y se asoció con todos los factores analizados. La probabilidad de insatisfacción fue mayor entre el sexo femenino (OR = 3,86; IC_95_% 3,45;4,32), tener internet en casa (OR = 8,68; IC_95_% 6,83;11,03), creer que nadie se preocupa por ellos (OR = 3,02; IC_95_% 2,60;3,50), que la vida no vale la pena (OR = 3,27; IC_95_% 2,88;3,72) y sentirse irritado (OR = 2,87; IC_95_% 2,53;3,26).

**Conclusión:**

La insatisfacción con la imagen corporal se asoció con varios factores y requiere un enfoque intersectorial.

## INTRODUCTION

Adolescence is a phase of life in which transformations occur that range from cognitive and biological to affective and social aspects and are directly related to lifestyle, habits, nutrition, physical activity, sexuality and corporeality as a whole. These physical and psychological changes can establish habits that last for the rest of a person’s life.^1^

An example of psychological change is body image, a representation that a person builds in their mind in relation to their own body, taking into account thoughts and feelings regarding its size, appearance and shape. Factors such as age, gender, psychological characteristics and self-esteem can influence perception of body image. Body image dissatisfaction can arise in childhood and lead to future mental disorders.^2^

The most common mental disorders are anxiety and depression, which can disrupt several aspects of an individual’s life, reducing reasoning, memorization and interest in the teaching-learning process.^3^ Furthermore, anxiety disorders are associated with a state of highly exhausting alertness, and depression is not just about mood changes, but also involves psychomotor, cognitive and vegetative changes.^4^

During adolescence, the changes that occur in the body play an important role in perception of body image, since, due to sociocultural standards and models imposed by the media, distortions and dissatisfaction can occur in the body image that adolescents have of themselves, leading to body-related attitudes that can harm their health and development.^5,6^

Such attitudes can lead to eating disorders, such as anorexia nervosa and bulimia nervosa. These are multifactorial diseases that are characterized by excessive concern about weight, with the possibility of severe dietary restrictions combined with distortion of body image, as well as a large compulsive intake of food in a short period of time, combined with a feeling of loss of control and inappropriate compensatory behaviors, such as inducing vomiting, for weight control.^7,8^

Studies on body image dissatisfaction generally only deal with the phenomenon associated with eating and mental health disorders, based on the understanding that food and drink are related to happiness or unhappiness with one’s body.^9,10^ However, given that perception of body image is influenced by several factors and that these can be modifiable, the present study investigated other important variables, such as relationship and routine coexistence with parents, level of physical activity and sedentary behavior, thus expanding knowledge about the issue. Furthermore, it is necessary to seek to understand the relationship between body image dissatisfaction and adolescence, as this is a period of transition and complex transformations in a person’s life, which can reflect on their development in adulthood and their social relationships.

In view of this, the objective of this study was to estimate prevalence and analyze factors associated with body image dissatisfaction among Brazilian adolescent school students.

## METHODS

This is a cross-sectional study using data from the National School Health Survey (*Pesquisa Nacional de Saúde do Escolar* - PeNSE), a population-based survey conducted in 2019 by the Brazilian Institute of Geography and Statistics (*Instituto Brasileiro de Geografia e Estatística* - IBGE), in partnership with the Ministry of Health, with the aim of collecting information about health of schoolchildren in Brazil and identifying risk and protective factors.^11^ The data, questionnaires and other documents are available to the public on the IBGE website.^11^ The data was accessed in August 2023.

The PeNSE 2019 survey included new indicators and its geographic coverage was expanded in relation to previous editions of the survey, so as to cover the country’s macro-regions, its Federative Units and state capital cities.^11^

The PeNSE sample was probabilistic involving clusters in two stages, whereby schools were selected in the first stage, and classes of enrolled students were selected in the second stage. The schools in the sample were selected with probabilities proportional to their size, measured by the number of classes reported in the 2017 School Census school registry. The classes were then selected randomly, with probability equal to the number of classes. Students present on the day of data collection were automatically included in the research. Information about the sampling plan is available in an IBGE publication.^11^

Data collection took place from April to September 2019, and the study population consisted of school students between 13 and 17 years old, enrolled and with regular attendance, attending both public and private elementary schools and high schools throughout Brazil.^11^

The dependent variable was self-reported “body image dissatisfaction”, derived by asking the following question: *How do you feel in relation to your body?* The answers were categorized into “satisfied” (satisfied, very satisfied) and “dissatisfied” (dissatisfied, very dissatisfied). The independent variables were organized into the categories listed below.

- Sociodemographic characteristics: sex (male, female); age (≤ 15 years, > 15 years); lives with mother (yes, no); number of people in the household (≤ 4 people, > 4 people); internet access at home (yes, no).
- Eating behavior: weekly frequency of having breakfast (≤ 4 days, > 4 days); weekly frequency of having lunch/dinner with guardian (≤ 4 days, > 4 days); weekly frequency of eating while doing other activities (≤ 4 days, > 4 days).
- Level of physical activity, based on total time per week spent on physical activities: inactive (< 300 hours/week); active (≥ 300 hours/week).
- Mental health: based on how they felt in the 30 days prior to answering the questionnaire, each adolescent study replied informing the frequency with which the felt worried about everyday activities, such as school activities, sports competitions, homework, amog others (never or rarely, almost always); frequency of thinking that no one cares about them (never or rarely, almost always); frequency of feeling irritated (never or rarely, almost always); frequency of thinking that life is not worth it (never or rarely, almost always).

Initially, descriptive analyses (absolute frequency and relative frequency/prevalence) of the sociodemographic variables were performed. Then, bivariate (crude) association analyses were performed using Pearson’s chi-square test and odds ratios (OR), with the aim of selecting independent variables for inclusion in the multivariate (adjusted) model. Factors significantly associated with body image dissatisfaction in the bivariate analysis were included in the multivariate model, considering a significance level of up to 0.25.^12^

We conducted multiple logistic regression (MLR) analysis to estimate the adjusted OR, as well as their 95% confidence intervals (95%CI). We used the *enter* method to carry out the analysis, which tests the model with all variables included. Significance of the adjusted OR was analyzed using their 95%CI. If the 95%CI included the value 1, this indicated that there is no association. With the exception of the absolute frequencies, all statistics and analyses took into account the complex sampling plan and test assumptions, including the multicollinearity test for multiple logistic regression. The analyses were carried out using the Statistical Package for Social Sciences version 20.0, using the Complex Samples Module procedures, used for data obtained through complex sampling plans. 

The PeNSE 2019 survey was approved by the National Research Ethics Committee of the National Health Council, as per Opinion No. 3.249.268. Students who agreed with and signed the Free and Informed Consent form took part in the research.^11^

## RESULTS

Of the total of 183,264 students enrolled and attending school, 159,245 students participated in the survey with valid questionnaires. The majority of participants were female (50.3%), 37.1% lived in the Southeast region of Brazil, almost half were between 16 and 17 years old (49.3%) and had were of mixed race/skin color (42. 5%). The majority lived with their mother (84.7%), in households with up to four people (71.5%), and had internet access at home (92.5%) (Table 1).

**Table 1:** Sociodemographic characteristics of adolescent school students according to the National School Health Survey-PeNSE, conducted between April and September 2019, Brazil, 2019

Variables	n (%)
**Sociodemographic characteristics**	
**Sex**	
Male	78,011 (49.7)
Female	80,788 (50.3)
**Region**	
North	35,792 (10.2)
Northeast	55,657 (27.7)
Southeast	28,377 (37.1)
South	17,192 (13.5)
Midwest	22,227 (11.5)
**Age**	
Under 13 years	25,642 (6.6)
13-15 years	82,389 (37.1)
16-17 years	42,509 (49.3)
18 years or over	8,276 (7.0)
**Lives with mother**	
Yes	141,468 (84.7)
No	17,687 (15.3)
**Number of people in household**	
≤ 4 people	105,667 (71.5)
> 4 people	53,450 (28.5)
**Internet access at home**	
Yes	144,380 (92.5)
No	14,796 (7.5)

Body image dissatisfaction prevalence was 30.2% (95%CI 29.2;31.1) (data not shown). The likelihood of body image dissatisfaction was higher in females (4.45, 95%CI 4.14;4.79), those aged over 15 years (1.41, 95%CI 1.31;1.52), who did not live with their mother (2.83; 95%CI 2.64;3.03), and had internet at home (4.10, 95%CI 3.33;5.04). We also found that adolescents who lived with more than four people in the same household had less likelihood of body image dissatisfaction (0.47; 95%CI 0.43;0.52). Regarding eating behavior, the odds of body image dissatisfaction increased significantly among adolescents who ate breakfast less frequently (2.15; 95%CI 1.98;2.33), had meals less frequently with their guardian (1. 78; 95%CI 1.66;1.91), and among those who ate while doing other activities for more than four days a week (1.81; 95%CI 1.63;1.94). Regarding mental health and level of physical activity, we found that the likelihood of adolescents being dissatisfied with their body image increased when they almost always or always felt worried about their everyday activities (2.04; 95%CI 1.93;2.15), thought no one cared about them (6.07; 95%CI 5.71;6.46), felt irritated (5.88; 95%CI 5.41;6.40) or thought that life is not worth it (10.04; 95%CI 9.21;10.93), and among adolescents whose level of physical activity fell into the inactive category (1.57; 95%CI 1.47;1.67 ) (Table 2).

**Table 2:** Crude association of satisfaction with body image and sociodemographic characteristics, eating behavior, level of physical activity and mental health among adolescent school students, Brazil, 2019

Variables	Satisfaction with body image		
	Yes (%)^a^	No (%)^a^	Crude OR (95%CI)^b^
Sociodemographic characteristics			
**Sex** ^c^			
Male	56,344 (84.5)	11,365 (15.5)	1.00
Female	44,993 (55.1)	26,430 (44.9)	4.45 (4.14;4.79)
**Age** ^c^			
≤ 15 years	70,524 (74.4)	24,164 (25.6)	1.00
> 15 years	30,810 (66.4)	13,642 (33.6)	1.41 (1.31;1.52)
**Lives with mother** ^c^			
Yes	90,632 (73.6)	33,354 (26.4)	1.00
No	10,947 (49.7)	4,532 (50.3)	2.83 (2.64;3.03)
**Number of people in household** ^c^			
≤ 4 people	65,706 (65.5)	26,596 (34.5)	1.00
> 4 people	35,854 (80.8)	11,286 (19.2)	0.47 (0.43;0.52)
**Internet access at home** ^c^			
Yes	90,790 (68.1)	35,563 (31.9)	4.10 (3.33;5.04)
No	10,797 (89.6)	2,329 (10.4)	1.00
**Eating behavior**			
**Frequency of having breakfast** ^c^			
≤ 4 days	34,324 (61.4)	19,483 (38.6)	2.15 (1.98;2.33)
> 4 days	67,271 (77.6)	18,407 (22.4)	1.00
**Has lunch/dinner with guardian** ^c^			
≤ 4 days	27,343 (60.7)	16,747 (39.3)	1.78 (1.66;1.91)
> 4 days	74,230 (73.5)	21,140 (26.5)	1.00
**Frequency of eating while doing other activities** ^c^			
≤ 4 days	49,204 (76.3)	15,462 (23.7)	1.00
> 4 days	52,318 (63.7)	22,420 (36.3)	1.81 (1.63;1.94)
**Level of physical activity** ^c^			
Inactive	69,987 (67.8)	29,513 (32.2)	1.57 (1.47;1.67)
Active	30,564 (76.2)	8,092 (23.8)	1.00
**Mental health**			
**Frequency of feeling worried about everyday activities, such as school activities, sports competitions, homework etc.** ^c^			
Never or rarely	51,443 (77.3)	11,494 (22.7)	1.00
Almost always or always	49,893 (62.3)	26,349 (37.7)	2.04 (1.93;2.15)
**Frequency of thinking that no one cares about them** ^c^			
Never or rarely	81,985 (81.4)	19,256 (18.6)	
			1.00
Almost always or always	19,384 (42.4)	18,595 (57.6)	6.07 (5.71;6.46)
**Frequency of feeling irritated** ^c^			
Never or rarely	69,628 (84.9)	14,587 (15.1)	
			1.00
Almost always or always	31,762 (49.0)	23,274 (51.0)	5.88 (5.41;6.40)
**Frequency of thinking that life is not worth it** ^c^			
Never or rarely	89,624 (78.1)	23,317 (21.9)	1.00
Almost always or always	11,653 (26.2)	14,520 (73.8)	10.04 (9.21;10.93)

a) Weighted percentage (sampling plan); b) Adjusted odds ratio and 95% confidence interval (95%CI) (sampling plan); c) Level of significance with p < 0.001 in Pearson’s chi-square test (crude association).

In the crude analysis, body image dissatisfaction showed statistically significant association with sociodemographic variables, eating behavior, level of physical activity and mental health (Table 2). In the adjusted analysis, except for the frequency of *feeling worried about everyday activities* variable, the other associations and significance remained. The likelihood of body image dissatisfaction was significantly higher for female adolescents (OR = 3.86; 95%CI 3.45;4.32), those aged over 15 years (OR = 1.43; 95%CI 1.28;1.60), who do not live with their mother (OR = 1.87; 95%CI 1.65;2.13) and who had internet at home (OR = 8.68; 95%CI 6.83;11.03). On the other hand, the likelihood of body image dissatisfaction was 52% lower among adolescents who lived in households with more than four people (OR = 0.48; 95%CI 0.42;0.54). In relation to eating behavior factors, the likelihood of body image dissatisfaction was significantly higher among adolescents who ate breakfast less frequently (OR = 1.96; 95%CI 1.69;2.27) and had lunch less frequently with their guardian (OR = 1.63; 95%CI 1.44;1.84), ate while doing other activities (OR = 1.56; 95%CI 1.42;1.71) and whose level of physical activity fell into the inactive category (OR = 1.82; 95%CI 1.56;2.12). Regarding mental health, the likelihood of body image dissatisfaction was greater among adolescents who thought that no one cared about them (OR = 3.02; 95%CI 2.60;3.50), felt irritated (OR = 2.87; 95%CI 2.53;3.26) or thought that life is not worth it (OR = 3.27; 95%CI 2.88;3.72) (Table 3). 

**Table 3:** Adjusted analysis of sociodemographic characteristics, eating behavior, level of physical activity and mental health associated with body image dissatisfaction among adolescent school students, Brazil, 2019

Variáveis	*Odds ratio* ajustada (IC_95%_)^a^
**Sociodemographic characteristics**	
**Sex**	
Male	1.00
Female	3.86 (3.45;4.32)
**Age**	
≤ 15 years	1.00
> 15 years	1.43 (1.28;1.60)
**Lives with mother**	
Yes	1.00
No	1.87 (1.65;2.13)
**Number of people in household**	
≤ 4 people	1.00
> 4 people	0.48 (0.42;0.54)
**Internet access at home**	
Yes	8.68 (6.83;11.03)
No	1.00
**Eating behavior**	
**Frequency of having breakfast**	
≤ 4 days	1.96 (1.69;2.27)
> 4 days	1.00
**Has lunch/dinner with guardian**	
≤ 4 days	1.63 (1.44;1.84)
> 4 days	1.00
**Frequency of eating while doing other activities**	
≤ 4 days	1.00
> 4 days	1.56 (1.42;1.71)
**Level of physical activity**	
Inactive	1.82 (1.56;2.12)
Active	1.00
**Mental health**	
**Frequency of feeling worried about everyday activities, such as school activities, sports competitions, homework etc.**	
Never or rarely	1.00
Almost always or always	1.07 (0.99;1.17)
**Frequency of thinking that no one cares about them**	
Never or rarely	1.00
Almost always or always	3.02 (2.60;3.50)
**Frequency of feeling irritated**	
Never or rarely	1.00
Almost always or always	2.87 (2.53;3.26)
**Frequency of thinking that life is not worth it**	
Never or rarely	1.00
Almost always or always	3.27 (2.88;3.72)

a) Adjusted odds ratio and 95% confidence interval (95%CI) – sampling plan.

## DISCUSSION

The research findings show that a set of factors contribute to adolescent body image dissatisfaction, both in the physiological aspect, being prevalent in the female sex, and in the relational and social aspect, such as eating habits and level of physical activity, internet access and family relationships. Also with regard to their mental health, body image dissatisfaction was more prevalent among adolescents who often felt worried about everyday activities, felt irritated and felt that life is not worth it, resulting in negative psychological results.

Body image dissatisfaction can prevail in both sexes, however, while girls generally want to have slimmer figures, boys prefer to be full-bodied.^13^ The greater likelihood of body image dissatisfaction demonstrated by girls over the age of 15 is due to the desire to adapt to the sociocultural standard focused on aesthetics, which sometimes does not match their reality. During this period, specific differences occur between the sexes, manifesting changes in the body. In females, body fat mass becomes more evident, and is generally concentrated more in the hips and thighs, while for males lean mass is more prevalent in their body composition. ^14^ In a study carried out with high school students from two public and four private schools in Rio de Janeiro on body image dissatisfaction, it was also predominant among females and those over 15 years old.^13^

Excessive social pressure for ideal bodies is reflected in the imposition of media culture, according to which the ideal woman must be slim, and the ideal man must be strong and athletic.^15^ This can have a negative psychological effect on adolescents, especially those who have internet access. For teenagers, due to ease of becoming addicted, technology can become a factor of social isolation, through use of the internet, and compromise their ability to socialize, making it difficult to distinguish between the real world and the virtual world, and how they perceive themselves in this context. In an integrative review, involving 32 articles from several countries – including the United States, Australia, England and Brazil –, covering the period from 2013 to 2018, the negative impact of social networks on body self-image of their subscribers was found, altering not only body image dissatisfaction levels, but also mood and self-esteem.^16^

A study carried out in Burkina Faso, an African country, in 2017, on eating disorders, body image and exposure to the media among adolescents, showed that this form of exposure causes an increase in body image dissatisfaction, which can consequently lead to eating disorders in the future.^17^ Inadequate eating behavior is a predictor of this issue, as teenagers who do not eat breakfast every day or eat while doing other activities (watching TV, using their cell phones, studying) tend to have greater body image dissatisfaction.

Just as good eating habits are important, so is having company while eating meals. Adolescents with lower frequency of having lunch/dinner with their father or mother are more likely to experience body image dissatisfaction. Another study^18^ had similar results and found that teenagers who regularly ate dinner with their parents were less likely to adopt harmful behavior in relation to their body in terms of weight control.^18^

Living with one’s family and having its support are important factors for preventing possible future harm to health.^19^ The very fact of not living with one’s mother or living with more than four people can be considered a predictor of body image dissatisfaction. This shows the importance of the family and the quality of these relationships for adolescent mental health. Family support and a good relationship with parents are very important for adolescents, as parents are psychosocial mediators and have a primary role in guidance and education, in addition to being positive and protective models for the developing individual.^19^ Closeness to parents is significantly related to body image satisfaction, emphasizing the importance of parental support for adolescents.^20,^ ^21^

Regarding effects on mental health, when worrying, irritability and feeling that no one cares about them increase, this may lead to adolescents distorting their body image, and this can cause symptoms of anxiety and depression. Symptoms of depression, if left untreated, can lead to depression itself, which increases the likelihood of body image dissatisfaction.^20^

Practicing regular physical activity, among other benefits, improves body composition and various physiological aspects that involve positive changes in relation to physical fitness and health as a whole.^22^ It is important for physical activity to be encouraged from a very early age, so that such changes positively influence the person’s development. On the other hand, physical inactivity can even affect people psychologically, as found in this study. Increasing the level of physical activity reduces depressive symptoms or possible depressive effects and, consequently, improves satisfaction with body image.^23^

The results of this study indicate that body image dissatisfaction is associated with several factors, which can pose serious risks to adolescents’ health and affect their biopsychosocial development. This issue deserves attention, especially when it comes to adolescence, a period of life in which the individual goes through several transformations that directly influence their development.

We suggest that projects be implemented in schools that encourage students to do physical activities, reduce screen time and have good eating habits, in addition to providing them with psychological support, in order to guide care for their mental health. School administrators must establish strategies to raise awareness among parents and guardians about the positive impact of good family relationships on their children’s body image satisfaction, consequently contributing to their good biopsychosocial development.

This study has limitations, as it had a cross-sectional design, estimating the relationships between the variables at just one moment in time, which does not allow causal relationships to be identified. Furthermore, as the questionnaires were self-administered, there is the possibility of information bias, which can influence the indicators we studied. However, the results of the study need to be taken into consideration, as they are nationally representative, in addition to its results being corroborated by other studies.^24,25,26^ Furthermore, this article is also a contribution to updating and encouraging new studies on this issue in Brazil. 

Our findings reinforce the importance of careful monitoring of the biopsychosocial aspect of adolescents, as well as investment in training of education professionals and actions that include the participation of professionals from a variety of sectors, in order to provide adolescents with education that addresses the biopsychosocial aspect, helping them to develop their autonomy, so that they are capable of acting in a manner that is both aware and beneficial for their lives. 
