# Supplementary material for: Prevalence and factors associated with body image dissatisfaction among school student: National School Health Survey, 2019
Source: Epidemiol Serv Saude. 2024 Dec 6;33:e20231441. doi: 10.1590/S2237-96222024v33e20231441.en (PMC11654045; doi:10.1590/S2237-96222024v33e20231441.en)
Supplement: Translation

# Translation: Prevalência e fatores associados à insatisfação com a imagem corporal de adolescentes escolares: Pesquisa Nacional de Saúde do Escolar, 2019

**DOI:** 10.1590/S2237-96222024v33e20231441.pt

**Authors:** Ingride Sousa Linhares, Osmar de Oliveira Cardoso, Jesusmar Ximenes Andrade, Fernando Ferraz do Nascimento, Malvina Thais Pacheco Rodrigues, Márcio Dênis Medeiros Mascarenhas

**Affiliations:** 1 Universidade Federal do Piauí, Programa de Pós-Graduação em Saúde e Comunidade, Teresina, PI, Brasil

**Keywords:** Adolescente, Imagem Corporal, Saúde, Escolas, Estudos Transversais

## Abstract

**Objetivo:**

Estimar a prevalência e analisar fatores associados à insatisfação com a imagem corporal de adolescentes escolares brasileiros.

**Métodos:**

Estudo transversal com dados da Pesquisa Nacional de Saúde do Escolar (PeNSE) de 2019. Estimou-se a prevalência de autorrelato da insatisfação com a imagem corporal e respectivos intervalos de confiança de 95% (IC_95_%) e sua associação com características individuais pela *odds ratio* (OR) e IC_95_% pela regressão logística.

**Resultados:**

Dos 159.245 escolares, 30,2% (IC_95_% 29,2;31,1) reportaram insatisfação com a imagem corporal, que se associou a todos os fatores analisados. A chance de insatisfação esteve mais relacionada a ser do sexo feminino (OR = 3,86; IC_95_% 3,45;4,32), possuir internet em casa (OR = 8,68; IC_95_% 6,83;11,03), achar que ninguém se preocupa com ele(a) (OR = 3,02; IC_95_% 2,60;3,50), que a vida não vale a pena (OR = 3,27; IC_95_% 2,88;3,72) e se sentir irritado(a) (OR = 2,87; IC_95_% 2,53;3,26).

**Conclusão:**

A insatisfação com a imagem corporal esteve associada a diversos fatores e requer uma abordagem intersetorial.

## Contribuições do estudo

**Principais resultados:**

A prevalência de insatisfação com a imagem corporal foi de 30,2%. A maior chance de insatisfação esteve mais relacionada a ser do sexo feminino, possuir internet em casa, sentir frequentemente que ninguém se preocupa com ele/ela, que a vida não vale a pena, e se sentir irritado(a).

**Implicações para os serviços:**

Sugere-se que sejam feitos projetos na escola incentivando a prática de atividade física, a diminuição do tempo de tela e bons hábitos alimentares, além de se fornecer suporte psicológico aos alunos, a fim de orientar cuidados com a sua saúde mental.

**Perspectivas:**

São necessários projetos de incentivo e instrução de boas práticas de saúde física e psicológica na escola, envolvendo toda a comunidade escolar, a formação de profissionais da educação e o incentivo ao autocuidado da saúde mental pelo adolescente.

## INTRODUÇÃO

A adolescência é uma fase da vida em que ocorrem transformações que vão dos aspectos cognitivos e biológicos aos afetivos e sociais e que se relacionam diretamente com o estilo de vida, hábitos, nutrição, atividade física, sexualidade e corporeidade como um todo. Essas alterações físicas e psicológicas podem estabelecer hábitos que perduram por toda a vida.^1^

Um exemplo de alteração psicológica é a imagem corporal, uma representação que o indivíduo constrói em sua mente em relação ao seu próprio corpo, levando em consideração pensamentos e sentimentos quanto ao seu tamanho, aparência e forma. Fatores como idade, sexo, características psicológicas e autoestima podem influenciar a percepção da imagem corporal. A insatisfação com a imagem corporal pode surgir ainda na infância e ocasionar psicopatologias futuras.^2^

As psicopatologias mais comuns são a ansiedade e a depressão, que podem atrapalhar diversos aspectos da vida do indivíduo, reduzindo a capacidade de raciocínio, memorização e interesse no processo ensino-aprendizagem.^3^ Além disso, os distúrbios de ansiedade estão associados a um estado de alerta altamente desgastante, e a depressão não se trata somente de alterações de humor, mas também de alterações psicomotoras, cognitivas e vegetativas.^4^

Na adolescência, as alterações que acontecem no corpo possuem um importante papel na percepção da imagem corporal, visto que, devido aos padrões socioculturais e a modelos impostos pela mídia, podem acontecer distorções e insatisfações na imagem corporal que esse adolescente tem de si mesmo, acarretando atitudes relacionadas ao corpo que podem prejudicar a sua saúde e seu desenvolvimento.^5,6^

Tais atitudes podem ocasionar transtornos alimentares, como a anorexia nervosa e a bulimia nervosa. Trata-se de doenças multifatoriais que se caracterizam pela preocupação excessiva com o peso, podendo haver uma severa restrição alimentar aliada à distorção da imagem corporal, bem como uma grande ingestão de alimentos compulsivamente em pouco tempo, aliada a uma sensação de perda de controle e comportamentos compensatórios inadequados, como a indução do vômito, para o controle de peso.^7,8^

Estudos sobre a insatisfação com a imagem corporal geralmente trazem o fenômeno apenas associado à alimentação e a transtornos na saúde mental, entendendo que a comida e a bebida estão relacionadas à felicidade ou infelicidade com o corpo que se tem.^9,10^ Contudo, compreendendo que a percepção da imagem corporal é influenciada por diversos fatores e que estes podem ser modificáveis, o presente estudo investigou outras variáveis importantes, como o relacionamento e a convivência rotineira com os pais, o nível de atividade física e o comportamento sedentário, ampliando o conhecimento sobre o tema. Além disso, a busca da relação entre a insatisfação com a imagem corporal e a adolescência se mostra necessária, visto que esse é um período de transição e complexas transformações na vida do indivíduo, que pode refletir na sua formação na fase adulta e nas suas relações sociais.

Diante do exposto, este estudo tem por objetivo estimar a prevalência e analisar fatores associados à insatisfação com a imagem corporal em adolescentes escolares brasileiros.

## MÉTODOS

Estudo transversal com dados da Pesquisa Nacional de Saúde do Escolar (PeNSE), inquérito de base populacional realizado em 2019 pelo Instituto Brasileiro de Geografia e Estatística (IBGE), em parceria com o Ministério da Saúde, com o objetivo de coletar informações acerca da saúde dos escolares do Brasil e identificar fatores de risco e de proteção.^11^ Os dados, questionários e outros documentos são de acesso público no *site* do IBGE.^11^ Os dados foram acessados em agosto de 2023.

Na PeNSE 2019, foram adicionados novos indicadores e a abrangência geográfica foi ampliada em relação às edições anteriores, que abrangeu grandes regiões, Unidades da Federação e municípios das capitais.^11^

A amostra da PeNSE foi probabilística por conglomerados em dois estágios, tendo sido as escolas selecionadas no primeiro estágio, e as turmas de alunos matriculados, no segundo. As escolas da amostra foram selecionadas com probabilidades proporcionais ao seu tamanho, medido pelo número de turmas informadas no cadastro de escolas do Censo Escolar 2017. Em seguida, as turmas foram selecionadas aleatoriamente, com probabilidade igual ao número de turmas. Os alunos presentes no dia da coleta dos dados foram automaticamente incluídos na pesquisa. Informações sobre o plano amostral podem ser obtidos em publicação do IBGE.^11^

A coleta dos dados foi realizada de abril a setembro de 2019, e a população de estudo foi constituída por escolares na faixa etária entre 13 e 17 anos, matriculados e com frequência regular, cursando os níveis de ensino fundamental e médio, em escolas públicas e privadas de todo o território nacional.^11^

A variável dependente foi a “insatisfação com a imagem corporal” autorreferida e obtida com base na pergunta: *Como você se sente em relação ao seu corpo?* As respostas foram categorizadas em “satisfeito” (satisfeito, muito satisfeito) e “insatisfeito” (insatisfeito, muito insatisfeito). As variáveis independentes foram organizadas nas categorias elencadas a seguir.

- Características sociodemográficas: sexo (masculino, feminino); idade (≤ 15 anos, > 15 anos); mora com a mãe (sim, não); número de pessoas na residência (≤ 4 pessoas, > 4 pessoas); tem internet em casa (sim, não).
- Comportamento alimentar: frequência semanal de realização do café da manhã (≤ 4 dias, > 4 dias); frequência semanal em que almoça/janta com o responsável (≤ 4 dias, > 4 dias); frequência semanal de comer fazendo outras atividades (≤ 4 dias, > 4 dias).
- Nível de atividade física, considerando-se o tempo total de atividade física semanal: inativo (< 300 h/sem.); ativo (≥ 300 h/sem.).
- Saúde mental: considerando o que foi sentido nos últimos 30 dias anteriores ao questionário, o adolescente respondia com que frequência se sentira preocupado com atividades cotidianas, como as escolares, competições esportivas, tarefas de casa, entre outras (nunca ou raramente, quase sempre); frequência de achar que ninguém se preocupa com ele/ela (nunca ou raramente, quase sempre); frequência de se sentir irritado(a) (nunca ou raramente, quase sempre); frequência de achar que a vida não vale a pena (nunca ou raramente, quase sempre).

Inicialmente, foram apresentadas análises descritivas (frequência absoluta e relativa/prevalência) apenas das variáveis sociodemográficas. Em seguida, foram realizadas análises de associação bivariada (bruta), por meio do teste qui-quadrado de Pearson e da razão de chances (*odds ratio* – OR), com o objetivo de selecionar as variáveis independentes para inclusão no modelo multivariado (ajustado). Foram incluídos no modelo multivariado os fatores significativamente associados à insatisfação com a imagem corporal na análise bivariada, considerando-se nível de significância de até 0,25.^12^

Foi conduzida análise de regressão logística múltipla (RLM) para estimar a OR ajustada, bem como seu intervalo de confiança de 95% (IC_95_%). Para a realização da análise, foi utilizado o método *enter*, que testa o modelo com todas as variáveis incluídas. A significância da OR ajustada foi analisada por meio do seu IC_95_%. Se o IC_95_% incluir o valor 1, isso indica que não há associação. Exceto pelas frequências absolutas, todas as estatísticas e análises levaram em conta o plano amostral complexo e os pressupostos dos testes, incluindo o teste de multicolinearidade para a regressão logística múltipla. As análises foram realizadas com uso do programa Statistical Package for Social Sciences versão 20.0, pelos procedimentos do Complex Samples Module, utilizado para dados obtidos por plano amostral complexo. 

A PeNSE 2019 foi aprovada na Comissão Nacional de Ética em Pesquisa do Conselho Nacional de Saúde, sob o parecer n^o^ 3.249.268. Participaram da pesquisa os alunos que concordaram e assinaram o Termo de Consentimento Livre e Esclarecido.^11^

## RESULTADOS

Do total de 183.264 alunos matriculados e com frequência, participaram da pesquisa 159.245 estudantes com questionários válidos. A maior parte dos participantes era do sexo feminino (50,3%), 37,1% eram moradores da região Sudeste e quase metade tinha de 16 a 17 anos de idade (49,3%). A maioria residia com a mãe (84,7%), em residências com até quatro pessoas (71,5%), e tinha acesso à internet em casa (92,5%) (Tabela 1).

**Tabela 1:** Características sociodemográficas dos adolescentes escolares segundo a Pesquisa Nacional de Saúde do Escolar-PeNSE, realizada entre os meses de abril e setembro de 2019, Brasil, 2019

Variáveis	n (%)
**Características sociodemográficas**	
**Sexo**	
Masculino	78.011 (49,7)
Feminino	80.788 (50,3)
**Região**	
Norte	35.792 (10,2)
Nordeste	55.657 (27,7)
Sudeste	28.377 (37,1)
Sul	17.192 (13,5)
Centro-Oeste	22.227 (11,5)
**Idade**	
Menos de 13 anos	25.642 (6,6)
De 13 a 15 anos	82.389 (37,1)
De 16 ou 17 anos	42.509 (49,3)
18 anos ou mais	8.276 (7,0)
**Mora com a mãe**	
Sim	141.468 (84,7)
Não	17.687 (15,3)
**N** **úmero** **de pessoas na residência**	
≤ 4 pessoas	105.667 (71,5)
> 4 pessoas	53.450 (28,5)
**Tem internet em casa**	
Sim	144.380 (92,5)
Não	14.796 (7,5)

A prevalência de insatisfação com a imagem corporal foi de 30,2% (IC_95_% 29,2;31,1) (dados não apresentados em tabelas). A chance de insatisfação com a imagem corporal foi mais elevada em indivíduos do sexo feminino (4,45, IC_95_% 4,14;4,79), com idade acima de 15 anos (1,41, IC_95_% 1,31;1,52), que não moravam com a mãe (2,83; IC_95_% 2,64;3,03), e com internet em casa (4,10; IC_95_% 3,33;5,04). Verificou-se também que adolescentes que moravam com mais de quatro pessoas tinham chance reduzida de insatisfação com a imagem corporal (0,47; IC_95_% 0,43;0,52). Quanto ao comportamento alimentar, a chance de insatisfação com a imagem corporal aumentou significativamente entre os adolescentes que tinham baixas frequências em tomar café da manhã (2,15; IC_95_% 1,98;2,33) e fazer refeições com o responsável (1,78, IC_95_% 1,66;1,91), e entre os que comiam fazendo outras atividades durante mais de quatro dias da semana (1,81; IC_95_% 1,63;1,94). Sobre a saúde mental e o nível de atividade física, foi observado que a chance de o adolescente estar insatisfeito com a imagem corporal aumentava quando quase sempre ou sempre se sentia preocupado com o dia a dia (2,04; IC_95_% 1,93;2,15), achava que ninguém se preocupava com ele/ela (6,07; IC_95_% 5,71;6,46), se sentia irritado(a) (5,88; IC_95_% 5,41;6,40) ou achava que a vida não vale a pena (10,04; IC_95_% 9,21;10,93), e entre os adolescentes de nível de atividade física inativo (1,57; IC_95_% 1,47;1,67) (Tabela 2).

**Tabela 2:** Associação bruta da satisfação com a imagem corporal e as características sociodemográficas, comportamento alimentar, nível de atividade física e saúde mental em adolescentes escolares, Brasil, 2019

Variáveis	Satisfação com a imagem corporal		
	Sim (%)^a^	Não (%)^a^	OR bruto (IC_95%_)^b^
Características sociodemográficas			
**Sexo** ^c^			
Masculino	56.344 (84,5)	11.365 (15,5)	1,00
Feminino	44.993 (55,1)	26.430 (44,9)	4,45 (4,14;4,79)
**Idade** ^c^			
≤ 15 anos	70.524 (74,4)	24.164 (25,6)	1,00
> 15 anos	30.810 (66,4)	13.642 (33,6)	1,41 (1,31;1,52)
**Mora com a mãe** ^c^			
Sim	90.632 (73,6)	33.354 (26,4)	1,00
Não	10.947 (49,7)	4.532 (50,3)	2,83 (2,64;3,03)
**N** **úmero** **de pessoas na residência** ^c^			
≤ 4 pessoas	65.706 (65,5)	26.596 (34,5)	1,00
> 4 pessoas	35.854 (80,8)	11.286 (19,2)	0,47 (0,43;0,52)
**Tem internet em casa** ^c^			
Sim	90.790 (68,1)	35.563 (31,9)	4,10 (3,33;5,04)
Não	10.797 (89,6)	2.329 (10,4)	1,00
**Comportamento alimentar**			
**Frequência de realização do café da manhã** ^c^			
≤ 4 dias	34.324 (61,4)	19.483 (38,6)	2,15 (1,98;2,33)
> 4 dias	67.271 (77,6)	18.407 (22,4)	1,00
**Almoça/janta com o responsável** ^c^			
≤ 4 dias	27.343 (60,7)	16.747 (39,3)	1,78 (1,66;1,91)
> 4 dias	74.230 (73,5)	21.140 (26,5)	1,00
**Frequência de comer fazendo outras atividades** ^c^			
≤ 4 dias	49.204 (76,3)	15.462 (23,7)	1,00
> 4 dias	52.318 (63,7)	22.420 (36,3)	1,81 (1,63;1,94)
**Nível de atividade f** **ísica** ^c^			
Inativo	69.987 (67,8)	29.513 (32,2)	1,57 (1,47;1,67)
Ativo	30.564 (76,2)	8.092 (23,8)	1,00
Saúde mental			
**Frequência de se sentir preocupado(a) com ocupações do dia a dia, como atividades da escola, competições esportivas, tarefas de casa etc.** ^c^			
Nunca ou raramente	51.443 (77,3)	11.494 (22,7)	1,00
Quase sempre ou sempre	49.893 (62,3)	26.349 (37,7)	2,04 (1,93;2,15)
**Frequência de achar que ninguém se preocupa com ele/ela** ^c^			
Nunca ou raramente	81.985 (81,4)	19.256 (18,6)	
			1,00
Quase sempre ou sempre	19.384 (42,4)	18.595 (57,6)	6,07 (5,71;6,46)
**Frequência de se sentir irritado(a)** ^c^			
Nunca ou raramente	69.628 (84,9)	14.587 (15,1)	
			1,00
Quase sempre ou sempre	31.762 (49,0)	23.274 (51,0)	5,88 (5,41;6,40)
**Frequência de achar que a vida não vale a pena** ^c^			
Nunca ou raramente	89.624 (78,1)	23.317 (21,9)	1,00
Quase sempre ou sempre	11.653 (26,2)	14.520 (73,8)	10,04 (9,21;10,93)

a) Percentual ponderado (plano amostral); b) *Odds ratio* e intervalo de confiança de 95% (IC_95_%) (plano amostral) ajustados; c) Nível de significância com p < 0,001 no teste qui-quadrado de Pearson (associação bruta).

 Na análise bruta, a insatisfação com a imagem corporal apresentou associação estatisticamente significativa com as variáveis sociodemográficas, de comportamento alimentar, do nível de atividade física e de saúde mental (Tabela 2). Na análise ajustada, exceto quanto à variável *frequência de se sentir preocupado com coisas do dia a dia*, as demais associações e a significâncias foram mantidas. A chance de insatisfação com a imagem corporal foi significativamente maior para adolescentes do sexo feminino (OR = 3,86; IC_95_% 3,45;4,32), com idade acima de 15 anos (OR = 1,43; IC_95_% 1,28;1,60), que não moram com a mãe (OR = 1,87; IC_95_% 1,65;2,13) e com internet em casa (OR = 8,68; IC_95_% 6,83;11,03). Entretanto, a chance de insatisfação com a imagem corporal foi 52% menor entre os adolescentes que residiam em domicílios com mais de quatro pessoas (OR = 0,48; IC_95_% 0,42;0,54). Em relação aos fatores de comportamento alimentar, a chance de insatisfação com a imagem corporal foi significativamente maior em adolescentes com baixas frequências de tomar café da manhã (OR = 1,96; IC_95_% 1,69;2,27) e de almoçar com o responsável (OR = 1,63; IC_95_% 1,44;1,84), comer fazendo outras atividades (OR = 1,56; IC_95_% 1,42;1,71) e com o nível de atividade física inativo (OR = 1,82; IC_95_% 1,56;2,12). No âmbito da saúde mental, a chance de insatisfação com a imagem corporal foi maior entre os adolescentes que achavam que ninguém se preocupava com eles (OR = 3,02; IC_95_% 2,60;3,50), sentiam-se irritados (OR = 2,87; IC_95_% 2,53;3,26) ou achavam que a vida não vale a pena (OR = 3,27; IC_95_% 2,88;3,72) (Tabela 3). 

**Tabela 3:** Análise ajustada das características sociodemográficas, comportamento alimentar, nível de atividade física e saúde mental associados à insatisfação com a imagem corporal em adolescentes escolares, Brasil, 2019

Variáveis	*Odds ratio* ajustada (IC_95%_)^a^
Características sociodemográficas	
**Sexo**	
Masculino	1,00
Feminino	3,86 (3,45;4,32)
**Idade**	
≤ 15 anos	1,00
> 15 anos	1,43 (1,28;1,60)
**Mora com a mãe**	
Sim	1,00
Não	1,87 (1,65;2,13)
**N** **úmero** **de pessoas na residência**	
≤ 4 pessoas	1,00
> 4 pessoas	0,48 (0,42;0,54)
**Tem internet em casa**	
Sim	8,68 (6,83;11,03)
Não	1,00
**Comportamento alimentar**	
**Frequência de realização do café da manhã**	
≤ 4 dias	1,96 (1,69;2,27)
> 4 dias	1,00
**Almoça/janta com o responsável**	
≤ 4 dias	1,63 (1,44;1,84)
> 4 dias	1,00
**Frequência de comer fazendo outras atividades**	
≤ 4 dias	1,00
> 4 dias	1,56 (1,42;1,71)
**Nível de atividade f** **ísica**	
Inativo	1,82 (1,56;2,12)
Ativo	1,00
**Saúde mental**	
**Frequência de se sentir preocupado(a) com ocupações do dia a dia, como atividades da escola, competições esportivas, tarefas de casa etc.**	
Nunca ou raramente	1,00
Quase sempre ou sempre	1,07 (0,99;1,17)
**Frequência de achar que ninguém se preocupa com ele/ela**	
Nunca ou raramente	1,00
Quase sempre ou sempre	3,02 (2,60;3,50)
**Frequência de se sentir irritado(a)**	
Nunca ou raramente	1,00
Quase sempre ou sempre	2,87 (2,53;3,26)
**Frequência de achar que a vida não vale a pena**	
Nunca ou raramente	1,00
Quase sempre ou sempre	3,27 (2,88;3,72)

a) *Odds ratio* ajustada e intervalo de confiança de 95% (IC_95_%) – plano amostral.

## DISCUSSÃO

Os achados da pesquisa mostram que um conjunto de fatores colaboram para a insatisfação da imagem corporal dos adolescentes, tanto no aspecto fisiológico, sendo prevalente no sexo feminino, quanto no relacional e social, como os hábitos alimentares e o nível de atividade física, acesso à internet e relacionamento familiar. Também no que diz respeito a sua saúde mental, sendo mais prevalente em adolescentes que frequentemente se sentem preocupados com as tarefas cotidianas, se sentiam irritados e com a sensação de que a vida não vale a pena, refletindo-se em resultados negativos no aspecto psicológico.

A insatisfação com a imagem corporal pode prevalecer em ambos os sexos, porém, enquanto as meninas geralmente querem ter silhuetas menores, os meninos preferem ter silhuetas maiores.^13^ A maior chance de insatisfação com a imagem corporal demonstrada pelas meninas na faixa de idade acima de 15 anos se deve ao desejo de adequação ao padrão sociocultural focado na estética, que às vezes não condiz com a sua realidade. Durante este período, ocorrem diferenças específicas entre os sexos, manifestando mudanças no corpo. Nas mulheres, a massa gorda corporal é mais evidente, e geralmente se concentra mais no quadril e nas coxas, enquanto nos homens a massa magra é mais prevalente em sua composição corporal.^14^ Em um estudo realizado com estudantes do ensino médio de duas escolas públicas e quatro privadas do Rio de Janeiro, para se verificar a insatisfação com a imagem corporal, também foram evidenciados predominância do sexo feminino e idade acima de 15 anos.^13^

A pressão social excessiva por corpos ideais se reflete na imposição da cultura midiática, segundo a qual a mulher ideal deve ser magra, e o homem, forte e atlético.^15^ Isso pode atingir negativamente o psicológico dos adolescentes, principalmente os que têm acesso à internet. Para o adolescente, devido a sua facilidade de adquirir um vício, a tecnologia pode se tornar um fator de isolamento social, por meio do uso da internet, e comprometer a sua capacidade de socialização, dificultando a distinção entre o mundo real e o mundo virtual, e como ele se percebe nesse contexto. Em uma revisão integrativa, realizada com 32 artigos de vários países – entre eles os Estados Unidos, a Austrália, a Inglaterra e o Brasil –, no período de 2013 a 2018, constatou-se a repercussão negativa das redes sociais na autoimagem corporal dos seus usuários, que alteraria não só os níveis de insatisfação com a imagem corporal, mas também o estado de humor e a autoestima.^16^

Um estudo realizado em Burkina Faso, um país da África, em 2017, sobre distúrbios alimentares, imagem corporal e exposição à mídia entre adolescentes, mostrou que essa exposição ocasiona aumento da insatisfação com a imagem corporal, o que consequentemente pode gerar desordens alimentares no futuro.^17^ O comportamento alimentar inadequado é um preditor para essa questão, visto que adolescentes que não tomam café da manhã todos os dias ou comem fazendo outras atividades (assistindo à TV, mexendo no celular, estudando) tendem a ter maior insatisfação com a imagem corporal.

Assim como bons hábitos alimentares são importantes, a companhia nessas refeições também. Adolescentes com uma frequência baixa de almoçar/jantar com o pai ou a mãe têm maior chance de insatisfação com a imagem corporal. Em outro estudo,^18^ também foram obtidos resultados semelhantes e se verificou que adolescentes que jantavam com os pais habitualmente apresentaram menor chance de adotar algum comportamento nocivo em relação ao seu corpo quanto ao controle de peso.^18^

A convivência e o suporte familiar são fatores importantes para a prevenção de possíveis prejuízos futuros à saúde.^19^ Só o fato de não morar com a mãe ou morar com mais de quatro pessoas pode ser considerado um preditor de insatisfação com a imagem corporal. Isso mostra a importância da família na saúde mental do adolescente, levando-se em consideração a qualidade das relações. O apoio familiar e a boa relação com os pais são muito importantes para o adolescente, pois os pais são mediadores psicossociais e têm papel primordial na orientação e educação, além de serem os modelos positivos e protetivos para o indivíduo em formação.^19^ A proximidade aos pais está significativamente relacionada à satisfação com a imagem corporal, enfatizando a importância do apoio parental para os adolescentes.^20,^ ^21^

Em relação aos reflexos na saúde mental, à medida que as preocupações, a irritabilidade e a sensação de que ninguém se preocupa com ele aumentam, o adolescente pode desencadear distorções na sua imagem corporal, e isso pode gerar sintomas de ansiedade e depressão. Sintomas de depressão, se não tratados, podem levar a quadros depressivos, o que aumenta a chance de insatisfação com a imagem corporal.^20^

A prática de atividade física regular, entre outros benefícios, melhora a composição corporal e diversos aspectos fisiológicos que envolvem modificações positivas em relação ao condicionamento físico e à saúde como um todo.^22^ É importante que essa prática seja incentivada desde muito cedo, para que as modificações influenciem no bom desenvolvimento do indivíduo. Caso contrário, a inatividade física pode afetar até o aspecto psicológico, como verificado neste estudo. O aumento do nível de atividade física diminui os sintomas depressivos ou possíveis efeitos depressivos e, por consequencia, melhora a satisfação com a imagem corporal.^23^

Os resultados deste estudo indicam que a insatisfação com a imagem corporal está associada a diversos fatores, o que pode trazer sérios riscos à saúde do adolescente e afetar o seu desenvolvimento biopsicossocial. Esse assunto merece atenção, principalmente quando se trata da fase da adolescência, período da vida em que o indivíduo passa por diversas transformações que influenciam diretamente a sua formação.

Sugere-se que sejam implementados projetos na escola que incentivem a prática de atividade física, a diminuição do tempo de tela e bons hábitos alimentares, além de fornecerem suporte psicológico aos alunos, a fim de orientar cuidados com a sua saúde mental. Os gestores escolares devem estabelecer estratégias para conscientizar pais e responsáveis acerca do impacto positivo da boa relação familiar na satisfação com a imagem corporal dos seus filhos, sendo-lhes proporcionado, por consequência, um bom desenvolvimento biopsicossocial.

Este trabalho apresenta limitações, visto que a pesquisa teve delineamento transversal, estimando as relações entre as variáveis em apenas um único momento, o que não permite identificar a relação causal. Além disso, por serem questionários autoaplicáveis, há possibilidade de viés de informação, o que pode influenciar os indicadores estudados. Todavia, seus resultados precisam ser levados em consideração, pois têm representatividade nacional, além de seus resultados serem ratificados em outros estudos.^24,25,26^ Além disso, o artigo também é uma contribuição para a atualização e o incentivo a novos estudos sobre essa temática no Brasil. 

Diante do que foi observado, reforça-se a importância de um acompanhamento atento do indivíduo no aspecto biopsicossocial, do investimento na capacitação de profissionais da educação e de ações que contemplem a participação de diversos profissionais, de modo a oferecer aos adolescentes uma formação que contemple o aspecto supracitado, auxiliando-os no desenvolvimento de sua autonomia, a fim de que sejam capazes de tomar atitudes conscientes e benéficas para suas vidas.
